# Case report: Hospital-acquired chickenpox in a healthcare setting

**DOI:** 10.1017/S0950268823001917

**Published:** 2023-12-19

**Authors:** Sandeepa Utpat, Nishka Utpat, Vinod Nookala, Lalitha Podakula, Kaanchi Utpat

**Affiliations:** 1 Faculty, Rutgers Health/Community Medical Center, Toms River, NJ, USA; 2 Independent Scholar, Internal Medicine, Infectious Diseases, Research Assistant at Rutgers Health/Community Medical Center, Toms River, NJ, USA; 3 Faculty, Rutgers Health/Community Medical Center, Toms River, NJ, USA; 4 Independent Scholar, Internal Medicine, Research Assistant at Rutgers Health/Community Medical Center, Toms River, NJ, USA

**Keywords:** chickenpox, varicella, epidemiology, varicella zoster virus (VZV), shingles, herpes zoster, nosocomial transmission, infection prevention

## Abstract

**Abstract:**

Chickenpox (varicella) is a rare occurrence in healthcare settings in the USA, but can be transmitted to healthcare workers (HCWs) from patients with herpes zoster who, in turn, can potentially transmit it further to unimmunized, immunosuppressed, at-risk, vulnerable patients. It is uncommon due to the inclusion of varicella vaccination in the recommended immunization schedule for children and screening for varicella immunity in HCWs during employment. We present a case report of hospital-acquired chickenpox in a patient who developed the infection during his prolonged hospital stay through a HCW who had contracted chickenpox after exposure to our patient’s roommate with herpes zoster. There was no physical contact between the roommates, but both patients had a common HCW as caregiver. The herpes zoster patient was placed in airborne precautions immediately, but the HCW continued to work and have physical contact with our patient. The HCW initially developed chickenpox 18 days after exposure to the patient with herpes zoster, and our patient developed chickenpox 17 days after the HCW. The timeline and two incubation periods, prior to our patient developing chickenpox, indicate transmission of chickenpox in the HCW from exposure to the herpes zoster patient and subsequently to our patient. The case highlights the potential for nosocomial transmission of chickenpox (varicella) to unimmunized HCWs from exposure to patients with herpes zoster and further transmission to unimmunized patients. Verification of the immunization status of HCWs at the time of employment, mandating immunity, furloughing unimmunized staff after exposure to herpes zoster, and postexposure prophylaxis with vaccination or varicella zoster immunoglobulin (Varizig) will minimize the risk of transmission of communicable diseases like chickenpox in healthcare settings. Additionally, establishing patients’ immunity, heightened vigilance and early identification of herpes zoster in hospitalized patients, and initiation of appropriate infection control immediately will further prevent such occurrences and improve patient safety.

**Summary:**

This is a case report of a varicella-unimmunized 31-year-old patient who developed chickenpox during his 80-day-long hospitalization. He had different roommates during his long hospital stay but had no physical contact with them and neither had visitors. On most days, the same HCW rendered care to him and his roommates. One of the patient’s roommates was found to have herpes zoster and was immediately moved to a different room with appropriate infection prevention measures. The HCW is presumably unimmunized to varicella and sustained significant exposure to the patient with herpes zoster during routine patient care which involved significant physical contact. The HCW was not furloughed, assessed for immunity, or given postexposure prophylaxis (PEP). The HCW had continued contact with our patient as part of routine care. On day 18, after exposure to the patient with herpes zoster, the HCW developed chickenpox. 17 days thereafter, our patient developed chickenpox. The time interval of chickenpox infection in the HCW after one incubation period after exposure to the patient with herpes zoster followed by a similar infection of chickenpox in our patient after another incubation period suggests the spread of varicella zoster virus (VZV) from the herpes zoster patient to the HCW and further from the HCW to our patient. Assessing the immunity of HCWs to varicella at the time of employment, ensuring only HCWs with immunity take care of herpes zoster and varicella patients, furloughing unimmunized exposed HCWs, offering PEP, and documentation of patients’ immunity to varicella at the time of hospital admission could help prevent VZV transmission in hospital settings. This is an attempt to publish this novel case due to its high educational value and relevant learning points.

## Introduction

Varicella, more commonly known as chickenpox, is caused by VZV, a herpes virus [1]. The virus attacks the immune and the nervous systems and, after a primary varicella infection, can reactivate later causing herpes zoster (shingles). Even though the disease is more common in children, chickenpox causes more severe disease in adults, pregnant women, and immunocompromised persons. The infection may start as a mild fever and malaise and tends to form itchy blisters and scabs [1]. The rash will develop on the face and torso and then gradually spread to the rest of the body, including legs, arms, and the scalp [2]. The contagious VZV that causes chickenpox spreads via direct contact and through inhalation of aerosols of the vesicular fluid of skin lesions from acute varicella or herpes zoster, and possibly through respiratory secretions that may be aerosolized [2]. Thus, unless the disease has already been developed or vaccination has been given, it is almost certain the disease will show up in the host.

Frequently, the immunization status of adult patients is not available or is incomplete [3]. In several states of the USA, although tested for immunity to varicella at the time of employment, HCWs are not required to get immunized to it [4]. Immunity to varicella should probably be mandated or non-mandatory strategies should be implemented in HCWs as they are in contact with patients who may be immunocompromised and therefore at risk of severe disease [ 5 ]. Immunizations of healthcare workers will help prevent the spread of communicable diseases such as varicella, as unvaccinated healthcare workers pose a risk of spreading such diseases to their colleagues and patients [6].

## Case presentation

**Figure 1.**
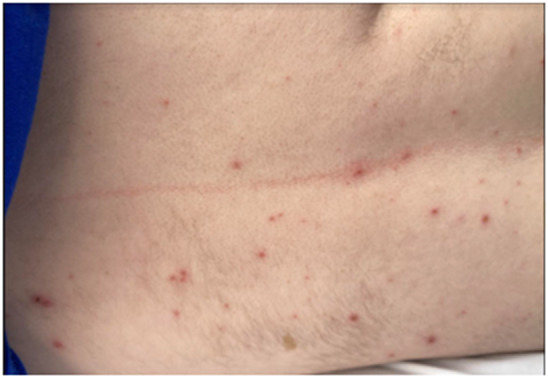
Maculopapular, vesicular, and pustular lesions with central necrosis and early crusting on the back.

**Figure 2.**
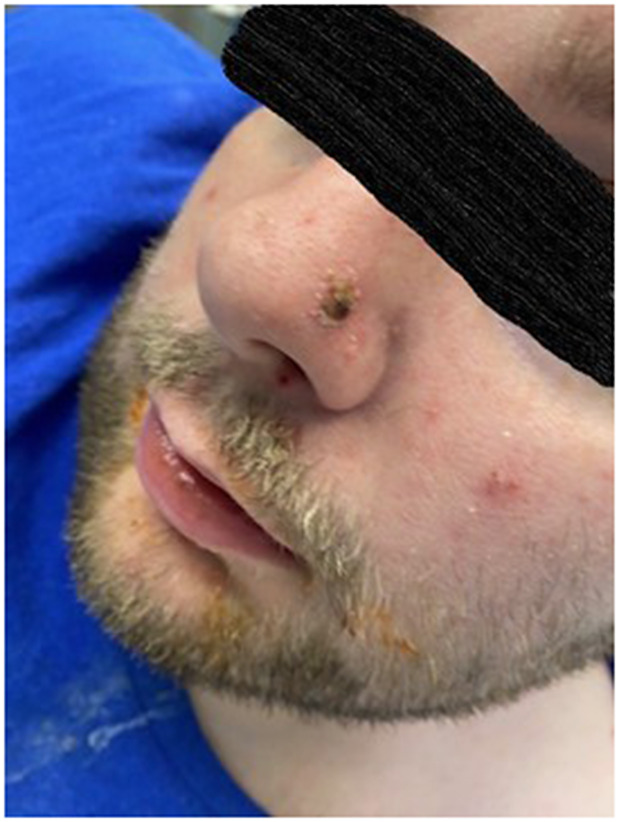
Crusted, vesicular lesion with central necrosis on the left nostril.

**Figure 3.**
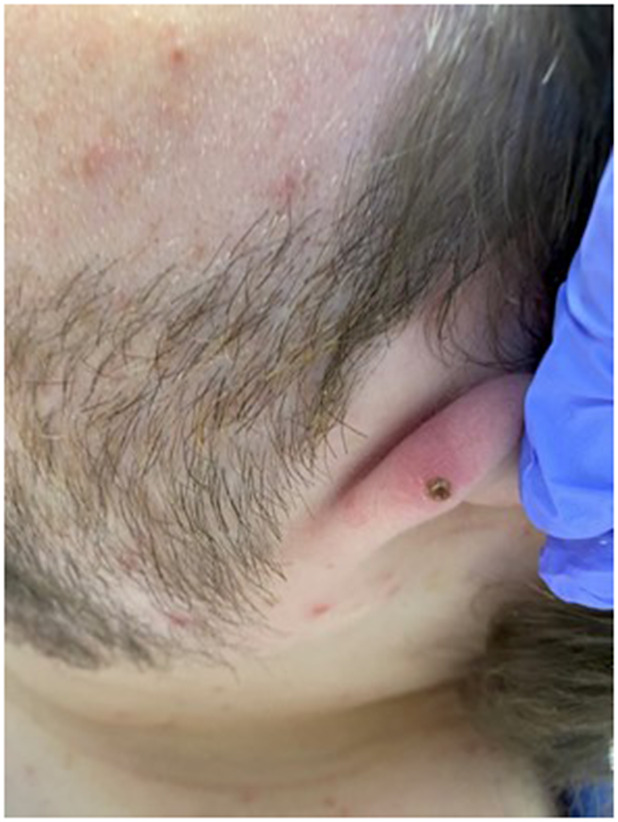
Crusted, vesicular lesion with central necrosis on the left ear lobe.

**Figure 4.**
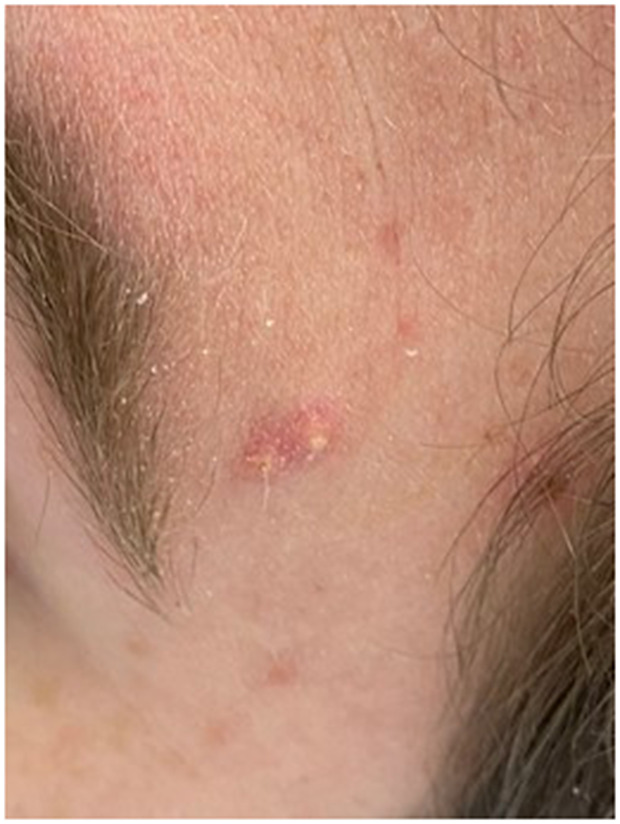
Pustular lesion on the left forehead.

**Figure 5.**
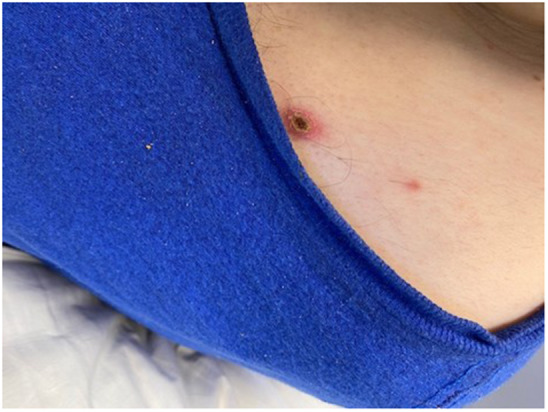
Macular lesion on the erythematous base with central necrosis on the left upper chest.

**Figure 6.**
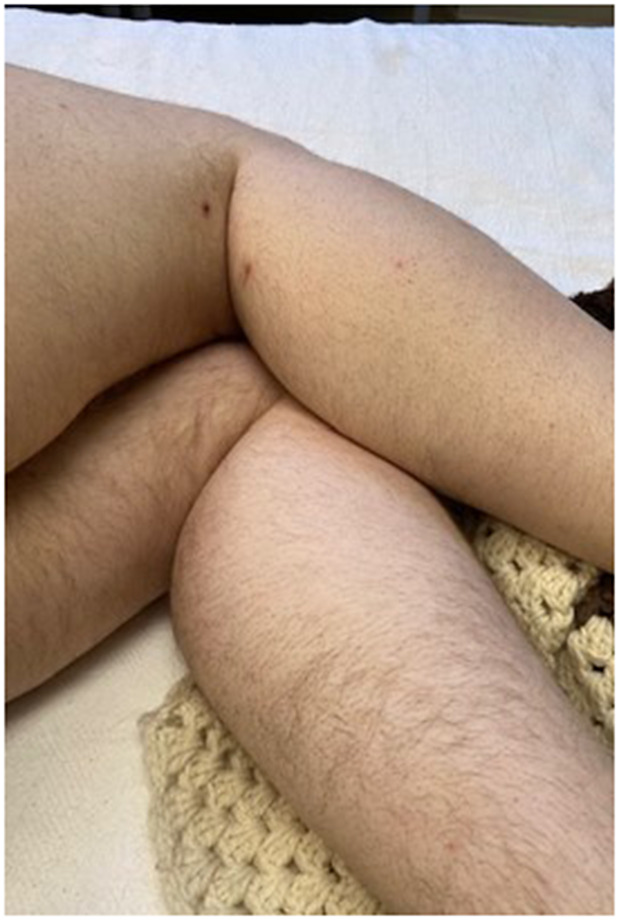
Macular lesions with early central necrosis on the posterior right lower extremity.

Here, we present a case of a 31-year-old intellectually disabled male patient, unimmunized for varicella, living previously with his elderly mother who functioned as his caregiver. He was hospitalized as he was unable to take care of himself when his mother was hospitalized. The patient then remained in the hospital for 80 days (from 10 January 2023 to 31 March 2023) due to challenges of a safe discharge. He was placed in a room with standard precautions which include hand hygiene, using gloves and surgical masks. He remained in the same room but had different roommates at various times. He had no physical contact with his roommates and neither had visitors. The same HCWs usually rendered care to him and his roommates. Around day 36, a HCW was exposed to one of his roommates with herpes zoster in lower lumbar dermatome. Although the lesions were in a covered area, the HCW had significant exposure due to close contact while rendering personal care to the patient. The roommate was immediately transferred to a different room with airborne precautions, but the HCW was not furloughed. The HCW developed varicella 18 days after that exposure, on day 54 of the patient’s hospitalization, and was furloughed thereafter with instructions to self-quarantine. 17 days afterwards, around day 71 of his hospital stay, the patient developed a low-grade fever, lack of appetite, and apathy (Tables 1:, 2:, 3). Subsequently, on or around day 73, staff noted the eruption of itchy, discrete, erythematous, pruritic, vesicular lesions on various parts of his body, suggestive of chickenpox (Figures 1, 2, 3, 4, 5 and 6). He was placed in airborne precautions immediately until the lesions scabbed off. He was noted to have eruption of vesicular lesions in small crops involving his face, torso and extremities. Roughly, he was noted to have 30 lesions. The lesions were initially vesicular, few became pustular and started to crust by days 5–6, and nearly all lesions had crusted and scabbed off by day 9. He did not have any complications of varicella and had complete recovery. Serology was requested on day 8 after the rash and showed elevated varicella IgM and varicella zoster IgG. (Table 4) As the patient was discharged to a group home on day 9, we did not have an opportunity to monitor his fourfold IgG titre after an appropriate period of time. The time interval of exposure of the HCW to the patient with herpes zoster and development of varicella herself was approximately one incubation period. The time interval between the HCW developing varicella and our patient developing varicella was one more incubation period. This timeline and evolution of events suggest the fact that the transmission occurred from the patient’s roommate with herpes zoster to the presumptive unimmunized staff and subsequently to our unimmunized patient, suggesting a breach of infection control measures.

**Table 1.** Complete blood count (CBC)

	Date				
Labs relative to the day of hospitalization	1/12 (Day 3)	1/21 (Day 11)	3/24 (Day 73)	Units	Reference range
WBC	71.1	4.6	3.4	×10^3^/mcL	4.80–10.80
RBC	5.09	4.76	5.01	×10^6^/mcL	4.20–5.40
HgB	12.2	11.5	12.2	g/dL	12.0–16.0
Hct	40.2	38.1	39.3	%	37.0–47.0
Plt	601	358	299	×10^3^/mcL	130–400

Abbreviation: g/dL, gram per decilitre; Hct, haematocrit; HgB, haemoglobin; mcL, microlitre; Plt, platelet; RBC, red blood cell count; WBC, white blood cell count.

**Table 2.** Vital signs

	Date		
Parameters relative to the onset of rash	3/22/23 (1 day prior to the onset of rash)	3/28/23 (5 days after the onset of rash)	Average normal range
Temperature (°F)	99.8 (H)	96.7	98.6
Systolic blood pressure (mmHg)	140	140	120
Diastolic blood pressure (mmHg)	70	84	80
Peripheral pulse rate (beats/min)	114 (H)	N/A	60–100
Respiratory rate (breaths/min)	20	18	12–16

**Table 3.** Viral test

Date	Test	Result
3/25/23 (2 days after the onset of rash)	Respiratory pathogen panel	Negative

**Table 4.** Serology

Date	Test	Result	Reference range
3/30/23 (7 days after the onset of rash)	Varicella IgM	2.67 (H)	< or = 0.90 = negative 0.91–1.09 = equivocal > or = 1.10 = positive
3/30/23 (7 days after the onset of rash)	Varicella zoster IgG	423.40	<135.00 = negative; no antibody detected 135.00–164.99 = equivocal > or = 165.00 = positive; antibody detected

## Discussion

The varicella vaccine was introduced in 1995 and recommended to all children before the age of 5 years and in non-immune adults [7]. It is recommended but not mandated for entry into schools and at the time of employment in healthcare settings. The Healthcare (formerly Hospital) Infection Control Practices Advisory Committee (HICPAC) has encouraged any facility or organization that provides direct patient care to formulate a comprehensive vaccination policy for all Health Care Provider (HCP) [8]. Hospitalization due to varicella in the USA has reduced >90% as a result of vaccination programmes, reducing nosocomial transmission in turn [9]. However, patients’ vaccination status and immunity to communicable diseases such as varicella are not routinely asked or recorded during hospitalizations. Records obtained afterwards from our patient’s primary care physician indicated that the patient was not vaccinated for varicella and he had never been to a formal school due to his profound developmental disability with behavioural disturbances and hence missed another opportunity to be vaccinated. At the time this case report was written, the vaccination or immunity status of the healthcare worker who developed chickenpox was not accessible to us due to privacy protection policy at the local level.

Assessing the varicella immunity of the HCW who had exposure to the herpes zoster patient, furloughing the exposed HCW, and offering PEP with varicella zoster immunoglobulin (VZIg) if the evidence of lack of immunity was found would have prevented the further transmission of varicella to our patient in this healthcare setting [9]. Developing a case definition by the facility’s Infection Prevention Department, contact tracing, identifying and vaccinating susceptible HCWs with two doses of varicella vaccine are additional measures to prevent nosocomial transmission [10]. Widely, the rash of uncomplicated chickenpox is accurately recognized by HCWs [11]. The recognition of rash in the HCW allowed immediate intervention and quarantine, thus preventing the HCW’s contribution to disease transmission from the hospital to the community. Furthermore, the involvement of the Infection Prevention Department of the hospital and consultation with Infectious Disease specialists after recognition of the rash in our patient prevented a potential outbreak on that particular floor and the hospital in general [12]. This event provided an opportunity to educate the hospital staff about herpes zoster and varicella, their potential for transmission, and the need to implement appropriate infection control measures in a timely manner [13, 15].

This raises the question of whether the practice of cohorting two different individuals in the same room with an unknown immunization status is rational. This also makes a case for individual rooms (solo rooms) for each hospitalized patient. The concerns of transmissibility between the roommates in hospitalized patients regardless of the 6 ft distance between the beds separated by a curtain, remain as they share the same healthcare workers assigned to their care. As HCWs are potentially responsible for the transmission of infections between two or more patients, establishing immunity of HCWs to vaccine-preventable communicable diseases like chickenpox (varicella) protects not only the individual HCW but also the patients who they render personal care to, thus having close contact with those patients [13]. Additionally, hospital-based HCWs have substantial physical contact with patients and differ significantly from those of community-based working adults, thus strengthening the need for immunity in HCWs to vaccine-preventable illnesses [14].

## Conclusion

This case demonstrates that vaccine-preventable diseases can be transmitted in a healthcare setting and can be prevented by introducing measures such as establishing policies to mandate varicella immunity in addition to assessing for it at the time of employment of HCWs. Unimmunized HCWs should be offered vaccinations at the time of employment. Vaccination and/or immunity to vaccine-preventable disease in patients at the time of hospital admission should be documented. This will allow patients’ placement in rooms with appropriate cohorts with a similar immunity status and assignment of HCWs appropriately to the patients with a similar immunity status. Periodic education that both herpes zoster and varicella are transmissible to susceptible patients and staff alike, and heightened awareness of healthcare workers are also of utmost importance in recognizing a vaccine-preventable disease. Hospitals have to weigh their professional responsibility to protect their patients from vaccine-preventable illnesses while developing initial employment policies for HCWs and subsequent measures required to be taken after exposure of HCWs to patients with herpes zoster and chickenpox.

## Data Availability

The data that support the findings of this study are openly available in StatPearls Publishing at https://www.ncbi.nlm.nih.gov/books/NBK448191/, reference 1. The data that support the findings of this study are openly available in InformedHealth.org at https://www.ncbi.nlm.nih.gov/books/NBK279621/, reference 2. The data that support the findings of this study are openly available in Human Vaccines and Immuno-therapeutics at https://pubmed.ncbi.nlm.nih.gov/33905303/, reference 3. The data that support the findings of this study are openly available in the Centers for Disease Control and Prevention at https://www2a.cdc.gov/vaccines/statevaccsApp/Administration.asp?statetmp=NJ#4, reference 4. The data that support the findings of this study are openly available in Hum Vaccin Immunother at https://pubmed.ncbi.nlm.nih.gov/26291642/, reference 5. The data that support the findings of this study are openly available in BMC Infectious Diseases at https://bmcinfectdis.biomedcentral.com/articles/10.1186/s12879-019-4,222-x, reference 6. The data that support the findings of this study are openly available in MMWR Morbidity Mortal Weekly Report at https://pubmed.ncbi.nlm.nih.gov/36757872/, reference 7. The data that support the findings of this study are openly available in MMWR Morbidity Mortal Weekly Report at https://www.cdc.gov/mmwr/pdf/rr/rr6007.pdf, reference 8. The data that support the findings of this study are openly available in Journal of Infectious Diseases at https://pubmed.ncbi.nlm.nih.gov/36265849/, reference 9. The data that support the findings of this study are openly available in Medical Journal Armed Forces India at https://pubmed.ncbi.nlm.nih.gov/35463537/, reference 10. The data that support the findings of this study are openly available in PLoS One at https://pubmed.ncbi.nlm.nih.gov/35749342/, reference 11. The data that support the findings of this study are openly available in Centers for Disease Control and Prevention at https://www.cdc.gov/infectioncontrol/guidelines/isolation/index.html, reference 12. The data that support the findings of this study are openly available in Vaccine at https://pubmed.ncbi.nlm.nih.gov/24726251/, reference 13. The data that support the findings of this study are openly available in Journal of Hospital Infection at https://doi.org/10.1016/j.jhin.2017.10.020, reference 14. The data that support the findings of this study are openly available in Journal of Korean Medical Sciences at https://pubmed.ncbi.nlm.nih.gov/30181734/, reference 15.
